# Predicting Brain Age of Healthy Adults Based on Structural MRI Parcellation Using Convolutional Neural Networks

**DOI:** 10.3389/fneur.2019.01346

**Published:** 2020-01-08

**Authors:** Huiting Jiang, Na Lu, Kewei Chen, Li Yao, Ke Li, Jiacai Zhang, Xiaojuan Guo

**Affiliations:** ^1^State Key Laboratory of Cognitive Neuroscience and Learning, Beijing Normal University, Beijing, China; ^2^College of Information Science and Technology, Beijing Normal University, Beijing, China; ^3^Banner Alzheimer's Institute, Phoenix, AZ, United States; ^4^Laboratory of Magnetic Resonance Imaging, The 306th Hospital of PLA, Beijing, China; ^5^Beijing Key Laboratory of Brain Imaging and Connectomics, Beijing Normal University, Beijing, China

**Keywords:** age prediction, convolutional neural networks, healthy subjects, machine learning, magnetic resonance imaging, structural network

## Abstract

Structural magnetic resonance imaging (MRI) studies have demonstrated that the brain undergoes age-related neuroanatomical changes not only regionally but also on the network level during the normal development and aging process. In recent years, many studies have focused on estimating age using structural MRI measurements. However, the age prediction effects on different structural networks remain unclear. In this study, we established age prediction models based on common structural networks using convolutional neural networks (CNN) with data from 1,454 healthy subjects aged 18–90 years. First, based on the reference map of CorticalParcellation_Yeo2011, we obtained structural network images for each subject, including images of the following: the frontoparietal network (FPN), the dorsal attention network (DAN), the default mode network (DMN), the somatomotor network (SMN), the ventral attention network (VAN), the visual network (VN), and the limbic network (LN). Then, we built a 3D CNN model for each structural network using a large training dataset (*n* = 1,303) and the predicted ages of the subjects in the test dataset (*n* = 151). Finally, we estimated the age prediction performance of CNN compared with Gaussian process regression (GPR) and relevance vector regression (RVR). The results of CNN showed that the FPN, DAN, and DMN exhibited the optimal age prediction accuracies with mean absolute errors (MAEs) of 5.55 years, 5.77 years, and 6.07 years, respectively, and the other four networks, i.e., the SMN, VAN, VN, and LN, tended to have larger MAEs of more than 8 years. With respect to GPR and RVR, the top three prediction accuracies were still from the FPN, DAN, and DMN; moreover, CNN made more precise predictions than GPR and RVR for these three networks. Our findings suggested that CNN has the optimal age prediction performance, and our age prediction model can be potentially used for brain disorder diagnosis according to age prediction differences.

## Introduction

Structural magnetic resonance imaging (MRI) studies have demonstrated that the brain undergoes profound age-related neuroanatomical changes during the normal development and aging process. These MRI studies have reported that the global gray matter volume linearly decreased with age, but the regional gray matter exhibited heterogeneous age effects (1–4). The gray matter volume in the frontal and parietal lobes and some regions of the temporal lobe substantially decreased with age, while some subcortical regions, such as the caudate and hippocampus, showed non-linear patterns (1, 3–8). More importantly, a number of structural MRI studies have found that the brain demonstrated age-related alterations at the network level (9–14). For example, the default mode network (DMN) exhibited significant differences in the gray matter volumes among healthy young, middle aged, and older subjects (9, 14). In recent years, many studies have focused on estimating brain age from structural MRI measurements. Such studies could advance our understanding of the relationship between brain morphometry and brain-predicted age (15).

Some common machine learning techniques have been widely used to build brain age prediction models in neuroscience, such as relevance vector regression (RVR), Gaussian process regression (GPR), and support vector regression (SVR) (16–21). Franke et al. used RVR method to estimate brain age based on the principal components of preprocessed gray matter images and obtained the mean absolute error (MAE) between the predicted and the chronological age of 5 years from healthy adult subjects (16). They also applied RVR to predict the brain ages of children and adolescents and estimated individual brain maturation using the age gap between the predicted age and real age (17). Additionally, Gaser et al. used the brain age gap estimate (BrainAGE) approach based on RVR from healthy subjects to more accurately identify the conversion of mild cognitive impairment (MCI) patients to Alzheimer's disease (AD) patients (18). Cole et al. trained a GPR model based on a similarity matrix that was formed by the vector dot-products from the normalized and concatenated gray and white matter images of healthy subjects aged 18–90 years and confirmed that this GPR model can accurately predict chronological age with an MAE of 5.02 years for the training dataset and 7.08 years for the test dataset from the Lothian birth cohort (21). Dosenbach et al. computed functional connections based on resting-state functional MRI (fMRI) data in typically developing participants aged from 7 to 30 years, then they used SVR to estimate the predicted ages which were converted to a functional connectivity maturation index (fcMI) to investigate brain functional maturity level and obtained accurate predictions about individuals' brain maturity across development (22). Liem et al. extracted the connectivity matrices and the cortical thickness, cortical surface area, and subcortical volumes as the feature vectors for resting-state fMRI and structural MRI, respectively, and constructed an age prediction framework using the single-source SVR as the first level and the multi-source random forest (RF) model as the second level of the age prediction analysis from a larger dataset of healthy subjects aged 19–82 years (19). Their results demonstrated that all models had good prediction performance with MAEs between 4.29 and 7.29 years and multimodal neuroimaging data improves brain age prediction performance (19).

In recent years, artificial neural network (ANN) with an input layer, an output layer and a single or limited hidden layer, which is referred to as neural network (NN), also has been used to build brain age prediction models. Lin et al. utilized ANN with one hidden layer to estimate age based on topological network properties from diffusion tensor imaging (DTI) of 112 healthy subjects aged 50.4–79.1 years and found that the predicted age was strongly related to the chronological age (*r* = 0.8) (23). Valizadeh et al. evaluated the age prediction effects on different anatomical feature sets of healthy subjects aged 7–96 years, such as cortical and subcortical volume, thickness, and area, using the following six machine learning techniques: multiple linear regression, support vector machine (SVM), ridge regression, NN with four hidden layers, RF, and k-nearest neighbor; they found that NN and SVM had the optimal age prediction accuracies among these six technologies (20). Though conventional machining learning methods including the shallow ANN performed well in age prediction, the feature extraction process is typically required in such machine learning. Feature extraction methods rely on manual designs based on prior professional knowledge, and thus have certain subjectivity and relatively poor generalization ability. Thus, the quality of the manually extracted features determines the performance of the whole prediction model to a large extent.

With the advancement of big data and improvements in computing infrastructure, more researchers switched their attention to deep learning models (24). Compared to conventional machining learning methods, deep learning methods can automatically learn abstract and complex features from brain imaging data using progressive non-linear transformations and have already been widely and successfully used in neuroimaging studies (25–29). Most of these deep learning-based studies investigated the neuroimaging correlations with brain disorders and explored aging and the prognostic or diagnostic biomarkers for healthy aging and brain disease diagnosis (25, 27, 28). The deep NN is the primary form of the present deep learning method in which convolutional neural network (CNN) is a representative deep network architecture. For example, Cole et al. utilized CNN to predict brain age from a large dataset of healthy subjects aged 18–90 years, and found that CNN had excellent age prediction performance with MAEs ranging from 4.16 and 4.65 years for normalized gray matter images using the diffeomorphic anatomical registration using exponential lie algebra (DARTEL) algorithm and raw structural MRI data with rigid registration and resampling to common voxel size and dimensions, respectively (25). Moreover, they suggested that the brain-predicted age represents an accurate, highly reliable and genetically influenced phenotype and can be potentially used as a brain aging biomarker (25).

Based on the structural covariance of gray matter morphology, researchers have found that different structural networks demonstrated age-related changes; however, the effects of brain age prediction on different structural networks remain unclear. In this study, we used the 3D CNN to establish brain age prediction models. First, we extracted 7 typical structural networks from structural MRI of 1,454 healthy individuals aged 18–90 years. Then, we separately trained and assessed the CNN-based brain age prediction model for each structural network. Last, we evaluated the performance of the CNN compared to GPR and RVR methods, respectively.

## Materials and Methods

### Subjects

All participants were from five publicly accessible databases: Autism Brain Imaging Data Exchange (ABIDE, https://fcon_1000.projects.nitrc.org/), Beijing Normal University (BNU, https://fcon_1000.projects.nitrc.org/), International Consortium for Brain Mapping (ICBM, https://www.loni.usc.edu/ICBM/), Information eXtraction from Images (IXI, https://brain-development.org/), and the Open Access Series of Imaging Studies (OASIS, https://www.oasis-brains.org/). These projects were approved by the local ethics committees. The whole data set used in this study consisted of 1,454 healthy individuals aged between 18 and 90 years (male/female: 713/741). The detailed information of all subjects is shown in Table 1. All subjects were free from major neurodegenerative or psychiatric diseases. All structural MRI data were acquired at either a 1.5 T or 3 T scanner using standard T1-weighted sequences. Written and informed consent was obtained for each participant at each local scanning site. The total subjects were randomly split into a training set (*n* = 1,303) and a test set (*n* = 151). The subjects' ages and genders were matched between these two data sets (*p* < 0.05).

**Table 1:** Information about the participants from the five datasets.

**Cohort**	**Number of subjects**	**Sex (male/female)**	**Age range (years)**	**Age mean (SD)**
ABIDE	172	152/20	18–56	26.04 (7.09)
BNU	198	76/122	18–26	21.16 (1.83)
ICBM	246	119/127	19–80	36.92 (14.08)
IXI	559	250/309	20–86	48.57 (16.49)
OASIS	279	116/163	18–90	44.95 (23.11)
Total	1454	713/741	18–90	39.51 (18.77)

### Data Preprocessing

All structural MRI data were preprocessed using the VBM8 toolbox (https://www.neuro.uni-jena.de/vbm8/) in the Statistical Parametric Mapping toolbox (SPM8, https://www.fil.ion.ucl.ac.uk/spm/) in MATLAB R2012b. The MRI images were segmented into gray matter, white matter, and cerebrospinal fluid with bias correction (30). Then, the gray matter maps were spatially normalized into the Montreal Neurosciences Institute (MNI) space using the DARTEL algorithm with final image matrix dimensions of 121 × 145 × 121 (31). The normalized gray matter images were multiplied by the Jacobian determinants with non-linear only modulation to preserve the absolute tissue volumes corrected for individual brain size. Finally, the modulated gray matter images were smoothed by an 8 mm full-width half-maximum Gaussian filter kernel. These smoothed gray matter images were prepared for the following analysis.

In this study, we chose the reference organization maps of the cerebral cortex from CorticalParcellation_Yeo2011 (https://surfer.nmr.mgh.harvard.edu/fswiki/CorticalParcellation_Yeo2011) (32) as the structural network templates. Yeo et al. applied a clustering approach to identify the parcellations of the functionally coupled regions in the human cerebral cortex according to the intrinsic functional connectivity from the fMRI of healthy adults. They divided the cerebral cortex into 7 networks in the MNI152 space: the frontoparietal network (FPN), the dorsal attention network (DAN), the DMN, the somatomotor network (SMN), the ventral attention network (VAN), the visual network (VN) and the limbic network (LN). They provided two kinds of parcellations: network liberal and tight masks. The cortical ribbon of the network liberal mask is defined in a more liberal fashion than that of the network tight mask, thus the network liberal mask covers more cerebral cortex regions than the tight mask does characterized by Yeo (32). As we consider aging impact on the brain will be broader and more brain regions will be with better prediction, we determined to use the seven network liberal masks. The overlapping regions of each subject's gray matter image and each network parcellation mask were defined as each corresponding structural network map.

### Correlation Analyses of Gray Matter

To explore the relationship between the gray matter of each structural network and age, we calculated the Pearson's correlation coefficient (*r*) between chronological ages and gray matter volumes of the structural network for each network. The total gray matter volume *v* was calculated by summing all the voxels within each structural network as the network's gray matter volume for each individual.

For each network, the *v* value was normalized to v^*^ using a min-max normalization method, calculated using the formula $v^{*}=\frac{v-min}{max-min}$, where *min* and *max* represent the minimum and maximum of gray matter volume of the corresponding network, respectively.

### Convolutional Neural Network Models

**Figure 1:**
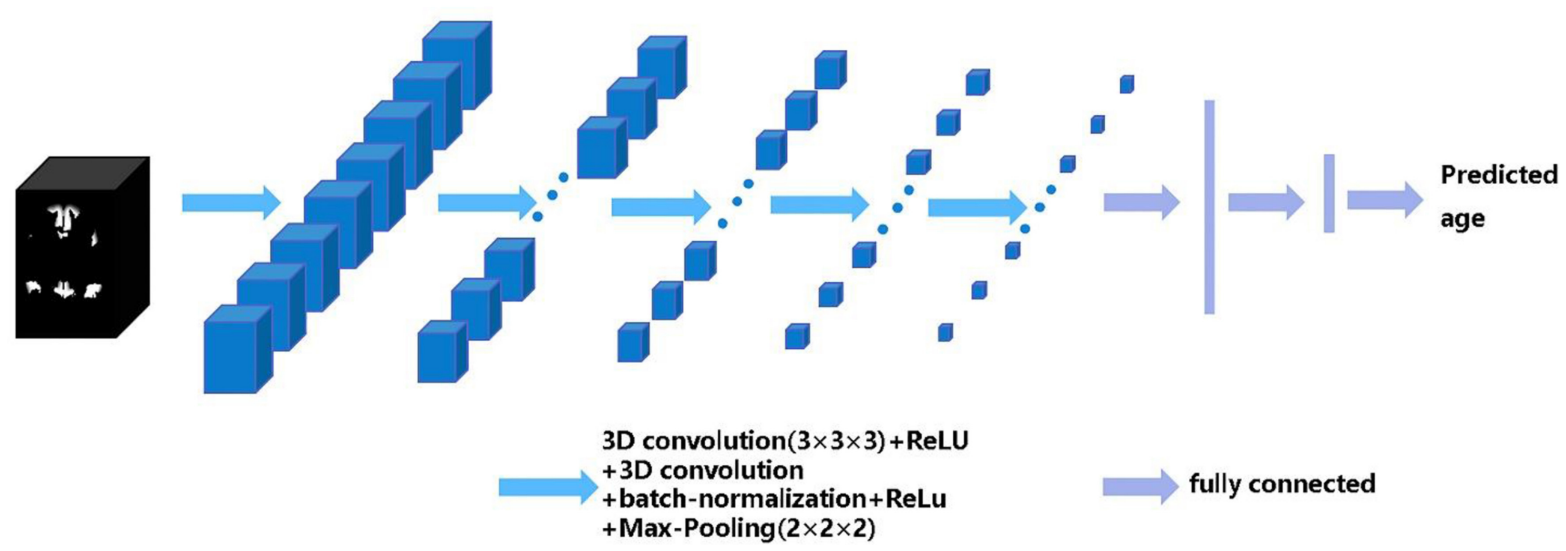
The architecture of the 3D convolutional neural networks (CNN). The black box represents the input structural network image and the blue boxes represent feature maps. The blue arrows indicate 3D CNN operations, the purple arrows indicate fully connected operations, the CNN model finally outputs the predicted age.

Our 3D CNN architecture was developed from VGG-13. Here, we replaced the 2D convolutional layer and 2D pooling layer in VGG-13 with a 3D convolutional layer and 3D pooling layer and added a batch normalization layer before the pooling layer (33). The inputs of each of our CNN models are all training samples' parcellation maps of each structural network. The output of the CNN model is the predicted brain age of each subject. The architecture of the CNN model includes five repeated stacks of a 3 × 3 × 3 convolutional layer (with a stride of 1 and padding of 1), followed by a rectified linear unit (ReLU) activation function, a 3 × 3 × 3 convolutional layer (with a stride of 1 and padding of 1), a 3D batch-normalization layer, a ReLU, a 2 × 2 × 2 max-pooling layer (with a stride of 2), and finally, three fully connected layers at the end. The number of channel maps in the convolutional layer of the first stack was set to eight and doubled after each max-pooling layer. The structure of CNN is shown in Figure 1.

We set the mini-batch size to 16, the learning rate to 0.01 with a constant decay of 0.1 after 10 epochs, the weight decay to 0.0005, the momentum to 0.9, and the number of epoch to 200. The convolutional kernel parameters of the CNN model are initialized using the He initialization method (34). Then, we optimized the parameters of the model using the mini-batch gradient descent with momentum and back propagation with the MAE function as the loss function during the training process. All codes were written in Torch7 using the Lua language and run on a GeForce GTX1080 Ti /PCIe/SSE2 GPU. We separately trained the CNN-based brain age prediction model for each structural network using the training set and predicted brain age of the subjects in the test set. Finally, we calculated *r*, goodness of fit (*R*^2^), MAE and root mean squared error (RMSE) between the chronological age and predicted age in the test dataset to evaluate the prediction performance of different brain structural networks.

### Gaussian Process Regression and Relevance Vector Regression

We estimated the brain age prediction performance of the CNN compared to the GPR and RVR methods, respectively. GPR is a non-parametric model that uses the prior of the Gaussian process in the regression analysis (35). Relevance vector machine (RVM), as the Bayesian alternative to SVM, can obtain sparse solutions based on the Bayesian model (36). RVM can get a probabilistic output and has a better generalization ability than SVM. When applying RVM to solve regression problems, we call the method RVR.

Both the GPR and RVR models were separately implemented for each structural network in the Pattern Recognition for Neuroimaging Toolbox (ProNTo V2.0, www.mlnl.cs.ucl.ac.uk/pronto) in MATLAB R2012b. We transformed each brain network image into a vector and obtained a subject (1454 healthy individuals) × voxel (the number of voxels in the brain network) feature matrix. Then, we applied a linear kernel function to the feature matrix and obtained a 1,454 × 1,454 similarity matrix. Finally, we separately built the GPR and RVR models using the chronological age as the dependent variable and the similarity matrix as the independent variable.

## Results

### Relationship Between Gray Matter Volume and Age

**Figure 2:**
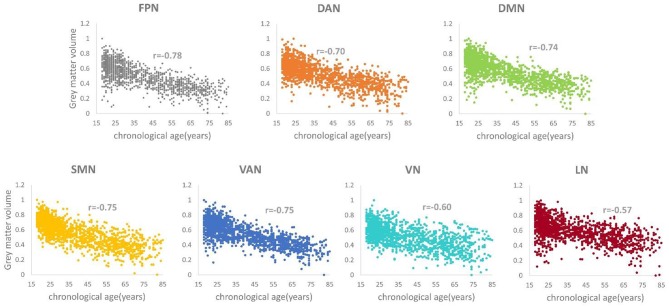
Correlation relationship between the gray matter volume and the chronological age for each structural network (*p*s < 0.05).

The *r* values between the gray matter volume of each brain network and the real age were −0.78, −0.70, −0.74, −0.75, −0.75, −0.60, and −0.57 (*p* < 0.05), respectively. The *r* values for the FPN, DAN, DMN, SMN, and VAN were more than or equal to seven, which means that there was a strong correlation between these five networks with age (Figure 2). In contrast to these five networks, the VN and LN had relatively weak correlations with age.

### Age Prediction Accuracy Using Convolutional Neural Networks

**Figure 3:**
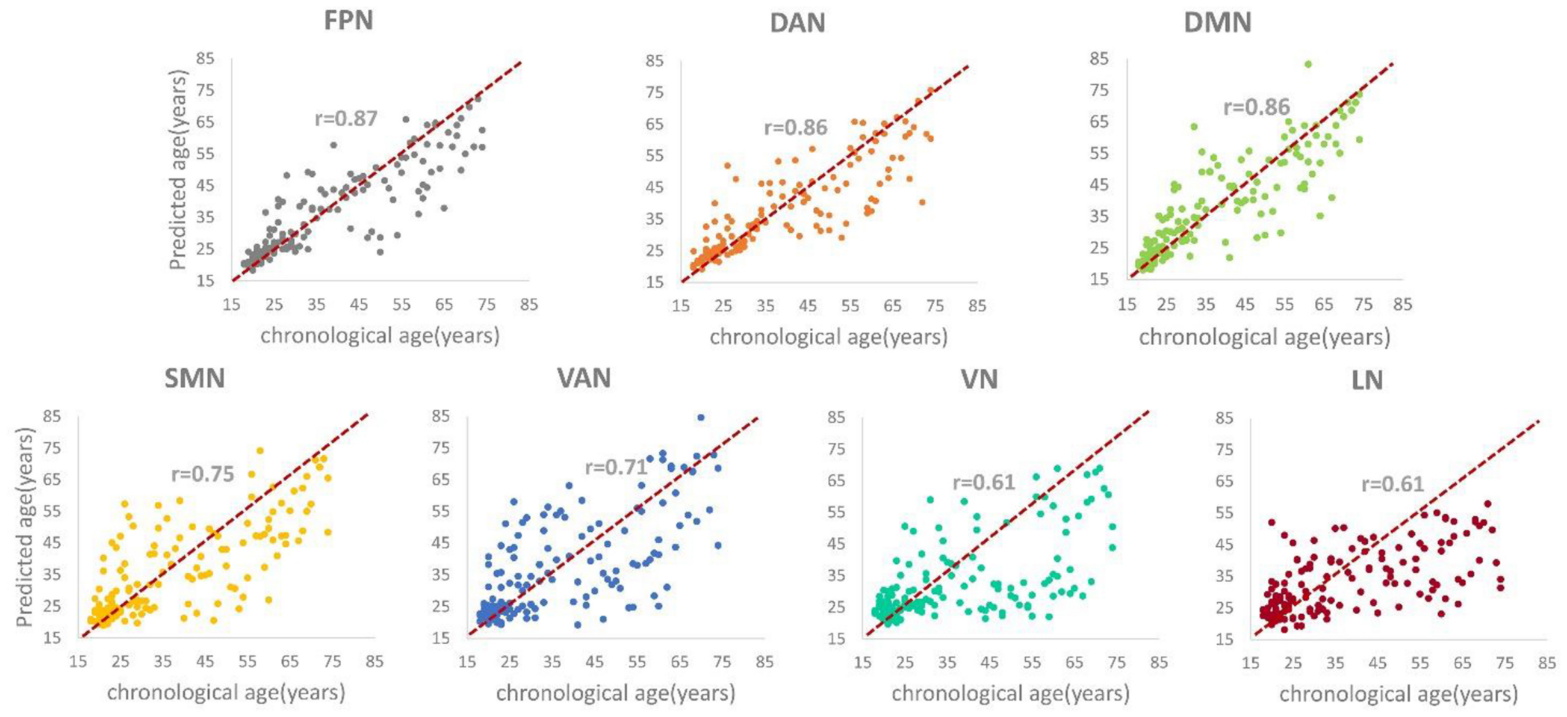
The scatter plots and correlation coefficients (*rs*) between the predicted brain age and the chronological age for each structural network (*p*s < 0.05).

**Figure 4:**
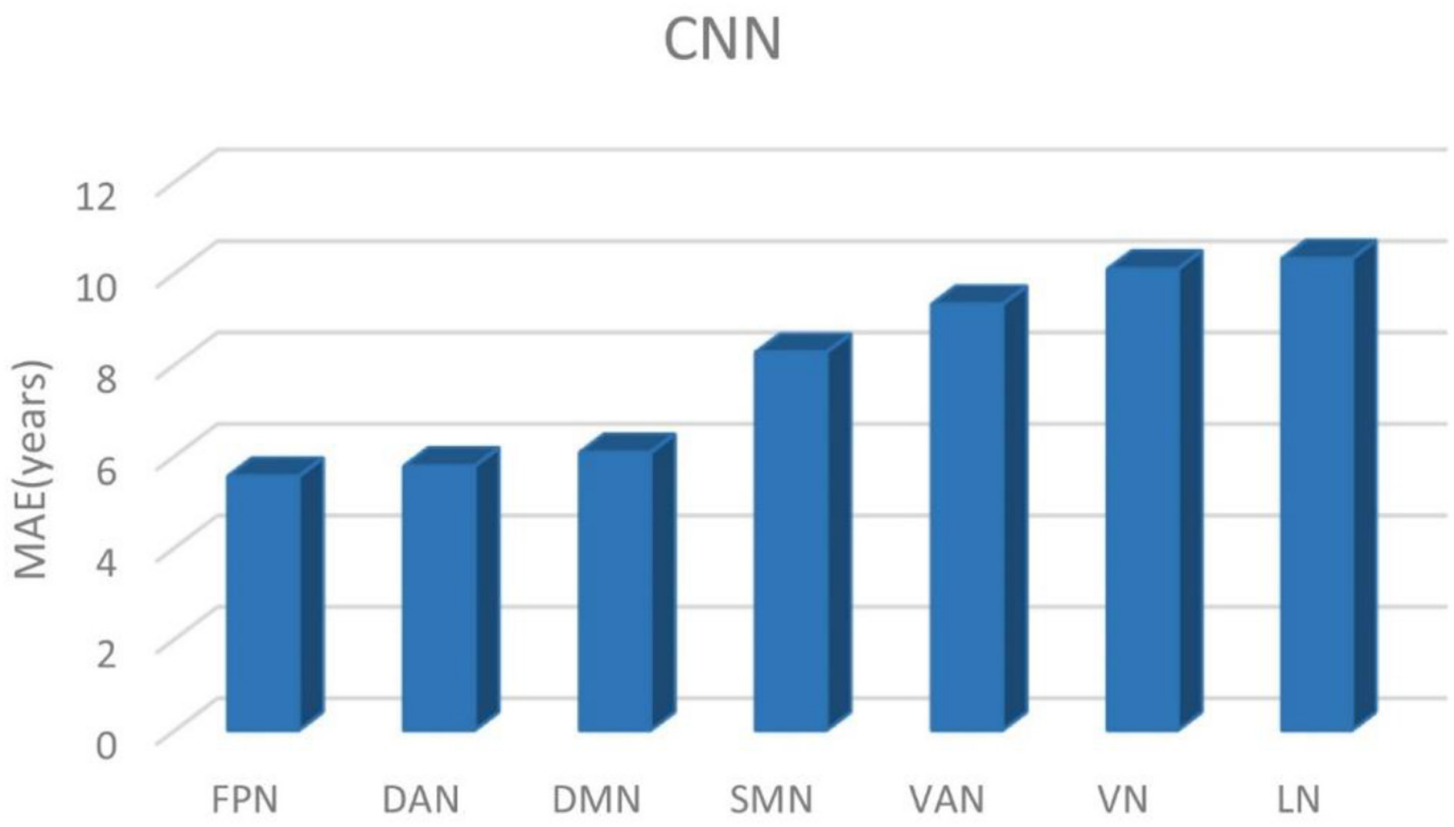
The brain age prediction MAEs using CNN for each structural network.

Table 2 shows the brain age prediction results of the seven brain structural networks using CNN. The table presents the *r*, *R*^2^, MAE and RMSE values between the chronological age and the predicted age in the test dataset. The MAEs of the CNN model for the seven structural networks were 5.55, 5.77, 6.07, 8.26, 9.31, 10.08, and 10.31, respectively. Among these networks, the FPN, DAN and DMN had better brain age prediction accuracy with *r*s of 0.87, 0.86, and 0.86, respectively, while the VN and LN performed worse with *r*s of 0.61 (Figures 3, 4). The *R*^2^s of the FPN, DAN and DMN were 0.76, 0.75, and 0.73, respectively, but the *R*^2^s of the VN and LN were 0.37 and 0.40, respectively. Moreover, the RMSEs of the FPN, DAN, and DMN were 8.37, 8.59, and 8.79, respectively, while the VN and LN obtained higher RMSEs of 14.21 and 13.96, respectively.

**Table 2:** Brain age prediction accuracy using CNN in the test dataset.

**Network**	**FPN**	**DAN**	**DMN**	**SMN**	**VAN**	**VN**	**LN**
*r*	0.87	0.86	0.86	0.75	0.71	0.61	0.61
*R*^2^	0.76	0.75	0.73	0.56	0.50	0.37	0.40
MAE (years)	5.55	5.77	6.07	8.26	9.31	10.08	10.31
RMSE	8.37	8.59	8.79	11.36	12.66	14.21	13.96

*r, Pearson's correlation coefficient; MAE, mean absolute error; RMSE, the root mean squared error; FPN, Frontoparietal network; DAN, Dorsal Attention network; DMN, Default mode network; SMN, Somatomotor network; VAN, Ventral Attention network; VN, Visual network; and LN, Limbic network*.

### Age Prediction Accuracy Using Gaussian Process Regression and Relevance Vector Regression

Table 3 presents the *r*, *R*^2^, MAE and RMSE values using GPR for the seven brain structural networks in the test dataset. The MAEs were 7.47, 7.86, 7.84, 8.24, 7.92, 8.13, and 8.35, respectively. With respect to the *r* values for the seven networks, the *r* values ranged from 0.80 to 0.84. The three networks, the FPN, DAN, and DMN, performed excellent among the seven networks (Table 3).

**Table 3:** Brain age prediction accuracy using GPR.

**Network**	**FPN**	**DAN**	**DMN**	**SMN**	**VAN**	**VN**	**LN**
*r*	0.84	0.81	0.82	0.80	0.81	0.80	0.80
*R*^2^	0.70	0.66	0.68	0.64	0.65	0.64	0.64
MAE (years)	7.47	7.86	7.84	8.24	7.92	8.13	8.35
RMSE	9.40	9.83	9.87	10.22	10.09	10.28	10.34

The MAEs of the RVR model for the seven structural networks reached 7.76, 8.04, 8.35, 8.51, 8.43, 8.57, and 8.88, respectively (Table 4). Furthermore, the *r* values were from 0.77 to 0.83, and the RMSEs were from 9.75 to 11.14.

**Table 4:** Brain age prediction accuracy using RVR.

**Network**	**FPN**	**DAN**	**DMN**	**SMN**	**VAN**	**VN**	**LN**
*r*	0.83	0.81	0.81	0.78	0.78	0.78	0.77
*R*^2^	0.68	0.66	0.65	0.61	0.61	0.61	0.59
MAE (years)	7.76	8.04	8.35	8.51	8.43	8.57	8.88
RMSE	9.75	9.93	10.41	10.73	10.65	10.84	11.14

**Figure 5:**
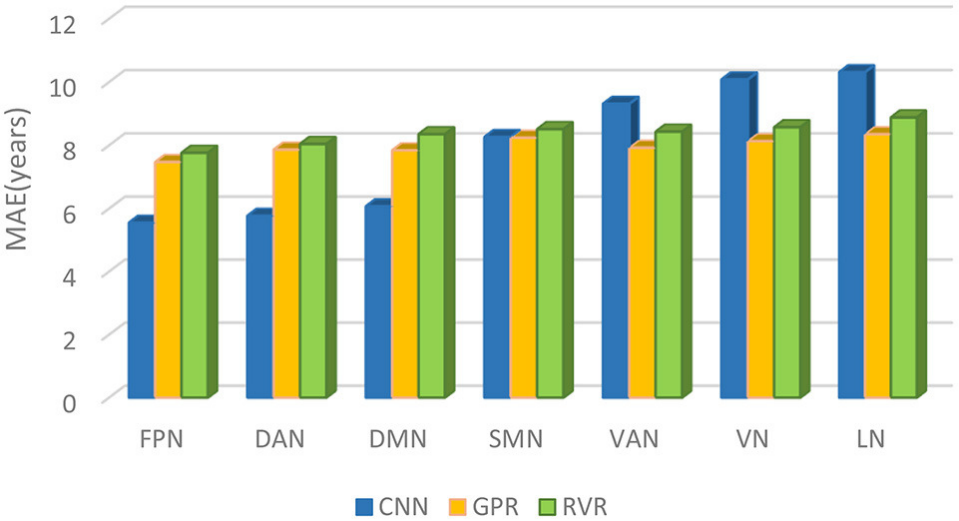
The brain age prediction MAEs of CNN, GPR, and RVR for each respective structural network.

Comparing CNN with GPR and RVR, Figure 5 depicted the age prediction accuracy MAEs for these three methods using a bar chart.

## Discussion

In this study, we built brain age prediction models based on seven brain structural networks using CNN and examined its prediction performance in comparison with GPR and RVR. The results showed that the seven structural networks exhibited similar prediction accuracy trends for the three methods of CNN, GPR, and RVR. The brain age prediction of the FPN, DAN, and DMN tended to be more accurate, and the other four networks, the SMN, VAN, VN, and LN, had the largest MAEs among the seven networks. The MAEs also showed that CNN has more precise prediction accuracy than that of GPR and RVR for the FPN, DAN, and DMN.

A large number of MRI studies have identified age-related structural covariation alterations from a between-group or lifespan perspective (9–14, 37–39). DuPre et al. applied seed partial least squares (PLS) to explore the life span trajectories of the gray matter volume covariance in the FPN, DAN, VN, SMN, VAN, and DMN from the structural MRI of healthy subjects aged 6–94 years and found two significant age-associated developmental trends: a stable pattern of structural covariance, whose integrity rapidly decreases across the lifespan, and an age-dependent pattern of structural covariance with an inverted *U*-shaped or linearly decreasing trajectory, with the exception of the VN (13). Using voxel-wise multiple regression analysis, Li et al. extracted eight seed regions of interest (ROIs) from gray matter volume images and investigated the age-related trajectories of structural covariance networks in healthy subjects aged 18–89 years (9). They found that all networks exhibited non-linear patterns with significant between-group differences in the language-related speech and semantic networks, the executive control network, and the DMN; they also found that fewer age-related changes existed in the SMN and VN for the young and middle to older groups (9). Spreng et al. reported that the structural covariance of the DMN declined in healthy and pathological aging processes (38). These studies showed that different brain networks demonstrated distinct age-related structural covariance patterns. The networks that are associated with high-order cognition, such as the FPN, DAN, and DMN, showed consistent significant age-dependent changes, but the networks that are associated with low-order cognition, such as the SMN and VN, experienced some discrepancies across the literatures. For example, DuPre et al. found that the SMN showed the age-dependent structural covariance trajectory as mentioned above, but the VN did not (13). Additionally, our previous study also demonstrated a significant age-related linear decrease for the SMN (39). However, Li et al.'s study reported little or no age-related alterations for the SMN and VN (9). In this study, our correlation analyses demonstrated that the gray matter volumes of all structural networks were negatively correlated with age, which means that the gray matter volumes of these brain networks declined with age (Figure 2). The changing trends in the degree of correlation, from the stronger to the weaker correlations, were basically consistent with the age-related findings of these structural networks in the literature.

The CNN-based results showed that different structural networks had different age prediction performances. Moreover, the age prediction accuracies of the different networks are basically consistent with the gray matter volume changing patterns with age. The FPN obtained the highest prediction accuracy among the seven structural networks with an MAE of 5.55 years, an *r* of 0.87, an *R*^2^ of 0.76 and an RMSE of 8.37. The FPN, DAN, and DMN can accurately predict the ages of healthy subjects, with the lowest MAEs of 5.55, 5.77, and 6.07 years, respectively. As a direct comparison, Cole et al. also used the CNN model to predict age based on structural MRI. Both their CNN architecture and ours contained the same repeated five blocks of convolution + ReLU, convolution + batch normalization + ReLU and max-pooling operations; however, their CNN had just one fully connected layer and ours had three fully connected layers at the end. Cole et al. obtained very good age prediction accuracies with the highest accuracy being MAE = 4.16 years for DARTEL normalized gray matter images and MAE = 4.65 years for rigid registrated structural MRI maps (25). Cole et al.'s CNN model extracted the features from the whole gray matter or structural MRI images while ours did it using structural networks containing some parcellations of gray matter. Nevertheless, the age prediction accuracies of our top three networks of the FPN, DAN and DMN were comparable to those of Cole et al. Additionally, a very recent study investigated brain age prediction based on whole brain voxel-wise functional connectivity patterns using CNN and obtained better prediction accuracy for resting-state fMRI, which indicated that the functional connectivity measures could be potential age prediction indices (40). The SMN and VAN exhibited moderate age prediction accuracies with MAEs = 8.26 and 9.31, respectively; however, the VN and LN had the lowest age prediction accuracies with MAEs = 10.08 and 10.31 years, respectively. The worst performance of the VN and LN may result from the non-significant age-related patterns.

The GPR-based and RVR-based results also demonstrated that the performance of the structural networks differed in their age prediction accuracy. The MAEs of the GPR-based model are 7.47, 7.86, and 7.84 years and the MAEs of the RVR-based model are 7.76, 8.04, and 8.35 years for the FPN, DAN, and DMN, respectively (Tables 3, 4). However, the MAEs of both the GPR-based model and RVR-based model are higher for the other four networks (the SMN, VAN, VN, and LN) than they are for these three networks. Then, GPR and RVR had consistent prediction trends with CNN for the seven brain networks. Compared with CNN, the top three prediction accuracies were still from the FPN, DAN, and DMN; moreover, CNN performed better than GPR and RVR for these three networks (Figure 5). For a more clear comparison, Table 5 lists the age prediction accuracies reported in literatures. Using RVR on the structural MRI data of healthy adults, Frank et al.'s study examined the influence of the preprocessing parameters, data reduction, regression method and training sample size on age prediction performance and got the lowest MAE of approximately 5 years using the principal components of gray matter images, which is better than the estimation accuracy of our RVR model but close to our best CNN-based results (16). Cole et al.'s GPR model was trained using the whole brain anatomical measures via concatenating normalized gray and white matter images, and obtained an MAE of 7.08 years for the independent test data (21). Additionally, with the best prediction accuracy of *R*^2^ = 0.84, Valizadeh et al. verified that the NN and SVM methods performed better on the combined feature set using the cortical thickness, volume and area than other machine learning methods, such as multiple linear regression, ridge regression, RF and k-nearest neighbors (20). We noted that it was hard to directly compare others results with ours due to the various structural MRI anatomical measures; however, our GPR and RVR models obtained comparable age estimation accuracy to these age prediction studies using traditional machine learning methods.

**Table 5:** Brain age prediction results reported in literatures.

**Articles**	**Modalties**	**Input data**	**Methods**	**Subjects (Age range)**	***r***	**MAE**	***R*^**2**^**
Cole et al. (21)	sMRI	GM+WM volumes	GPR	2001 NC (18–90)	0.94	5.02	0.88
Cole et al. (25)	sMRI	GM volume map Raw MRI map	CNN	2001 NC (18–90)	0.96 0.94	4.16 4.65	0.92 0.88
Franke et al. (17)	sMRI	GM+WM volumes	RVR	394 NC (5–18)	0.93	1.10	-
Franke et al. (16)	sMRI	GM volume	RVM	655 NC (19–86)	0.92	5.00	-
Li et al. (40)	rs-fMRI	Functional connectivity	CNN	983 NC (8–22)	0.61	2.15	
Liem et al. (19)	rs-fMRI + sMRI	Functional connectivity;Structural measures	SVR+RF	2354 NC (19–82)	-	4.29	-
Lin et al. (23)	DTI	Topological network properties	ANN	112 NC (50–79)	0.80	4.29	-
Valizadeh et al. (20)	sMRI	Anatomical feature sets	SVM NN	3144 NC (7–96)	-	-	0.84 0.84

*sMRI, Structural Magnetic Resonance Imaging; rs-fMRI, resting-state Functional Magnetic Resonance Imaging; DTI, Diffusion Tensor Imaging; GM, Gray Matter; WM, White matter; Raw MRI, rigid only registrated structural MRI; Structural measures: cortical thickness, cortical surface area, and subcortical volumes; Anatomical feature sets: cortical volume, thickness, area, subcortical volume, cerebellar volume, etc; GPR, Gaussian Process Regression; CNN, Convolutional Neural Network; RVR, Relevance Vector Regression; RVM, Relevance Vector Machine; SVR, Support Vector Regression; RF, Random Forest; ANN, Artificial Neural Network; SVM, Support Vector Machine; NN, Neural Network; NC, normal controls*.

With the super powerful parallel computing ability of GPUs, CNN models such as VGG, GoogLeNet and ResNet have exhibited excellent performance in predicting clinical neurodevelopment, neurodisease classification and age (25–29, 41, 42). The typical CNN architecture mainly consists of an input layer, a convolutional layer, a pooling layer and an output layer (43). In our study, based on VGG-13, we built the CNN model with five repeated stacks of a convolutional layer followed by an activation function ReLU, a convolutional layer, a batch-normalization layer, a ReLU and a max-pooling layer. CNN can learn characteristics from low-level common features to high-level complex features using multiple hidden layers from imaging data without a feature extraction process. Moreover, CNN takes advantage of local connections, shared weight parameters, and pooling, which reduce the network complexity and the number of training parameters, thus making it easier to train and providing it strong robustness and generalizability. With respect to the input data for GPR or RVR, the brain network image of each subject was transformed into a vector and further mapped into a similarity matrix. This procedure may lead to losing some important information of brain structural networks. We replaced the 2D CNN with the 3D CNN to comprehensively utilize the 3D brain informative regional or neighborhood correlated relationship among the voxels within structural networks. Compared with GPR and RVR, CNN obtained more accurate prediction results for the FPN, DAN, and DMN with the top three prediction accuracies.

Though CNN performed better in age prediction, similar to other deep learning research, it is hard to distinctly explain the learned models due to the black box characteristics of deep learning models. The brain network attributes and changing patterns that are identified by CNN may be used as biomarkers for brain aging. Additionally, the participants in this study are from 5 publicly-available databases which used different scanners, magnetic strength and acquisition protocols. This inter-scanner technical variability may introduce some uncertainty in the anatomical measure analysis for our study and others using multi-scanner data. Our spatial pre-processing steps included bias correction, segmentation, DARTEL normalization and smooth, which may accounts for some MRI inhomogeneity and noise from different scanners (25, 44). Further validation is necessary to examine systemic biases on the predicted brain age owing to inter-scanner technical differences when pooling multiple center data. A further limitation of this study is that we just considered the adult sample aged from 18 years. There is still a lack of massive MRI data from children to adolescents. The brain undergoes remarkable age-related brain alterations from childhood to adolescence, and research on a wider age range can provide age prediction results across the whole lifespan. Finally, gray matter alterations are known to play a major role in the brain development and aging process, especially the gray matter exhibits age-related network changes. We therefore used structural MRI networks as brain characteristics to predict age, but we only considered seven cerebral cortex networks. The number of networks is relatively smaller but these networks are more inclusive with regard brain cortical regions. We will consider to explore the age prediction effects on subcortical networks or fine-grained subnetworks in future study. It should be noted that multimodal imaging data can offer sufficient informative features that may improve the age prediction accuracy. However, some of five databases we used in this study lacked DTI or fMRI data. Research is currently underway on building a multi-channel CNN with functional connectivity from resting-state fMRI and structural connectivity from DTI data.

In the current study, we trained the age prediction models based on CNN for seven structural networks of healthy adults and evaluated the age estimation performance of CNN compared to GPR and RVR. Three structural networks (FPN, DAN, and DMN) provided the optimal age prediction accuracies, which suggested that these networks had significant age-related changes across the lifespan. Our findings also showed that CNN is superior to GPR and RVR for these three networks in age prediction. Research on age prediction has shed insight on understanding the relationship between age and the brain morphometrics in the human developmental and aging process. The prediction gap between the predicted age and chronological age represents the abnormal brain changes due to some neurodegenerative diseases. Our age prediction model based on CNN can be potentially used for brain disorder diagnosis using the age prediction differences.

## Data Availability Statement

All participants were from five publicly accessible databases: Autism Brain Imaging Data Exchange (ABIDE, https://fcon_1000.projects.nitrc.org/), Beijing Normal University (BNU, https://fcon_1000.projects.nitrc.org/), International Consortium for Brain Mapping (ICBM, https://www.loni.usc.edu/ICBM/), Information eXtraction from Images (IXI, https://brain-development.org/), and the Open Access Series of Imaging Studies (OASIS, https://www.oasis-brains.org/).

## Ethics Statement

The studies involving human participants were reviewed and approved by the local ethics committees. All participants were from publicly accessible databases. The patients/participants provided their written informed consent to participate in this study.

## Author Contributions

HJ, LY, and XG designed the study. HJ and NL performed data analysis. HJ, KC, KL, JZ, and XG interpreted the results. HJ, NL, and XG drafted the manuscript.

### Conflict of Interest

The authors declare that the research was conducted in the absence of any commercial or financial relationships that could be construed as a potential conflict of interest.
